# Nanoparticles for Cancer Therapy: Current Progress and Challenges

**DOI:** 10.1186/s11671-021-03628-6

**Published:** 2021-12-05

**Authors:** Shreelaxmi Gavas, Sameer Quazi, Tomasz M. Karpiński

**Affiliations:** 1 Department of Life Sciences, GenLab Biosolutions Private Limited, Bangalore, Karnataka 560043 India; 2 GenLab Biosolutions Private Limited, Bangalore, Karnataka 560043 India; 3 Chair and Department of Medical Microbiology, Poznań University of Medical Sciences, Wieniawskiego 3, 61-712 Poznań, Poland

**Keywords:** Cancer, Nanoparticles, Chemotherapy, Cellular targeting, Multidrug resistance, Cryosurgery, Scale-up

## Abstract

Cancer is one of the leading causes of death and morbidity with a complex pathophysiology. Traditional cancer therapies include chemotherapy, radiation therapy, targeted therapy, and immunotherapy. However, limitations such as lack of specificity, cytotoxicity, and multi-drug resistance pose a substantial challenge for favorable cancer treatment. The advent of nanotechnology has revolutionized the arena of cancer diagnosis and treatment. Nanoparticles (1–100 nm) can be used to treat cancer due to their specific advantages such as biocompatibility, reduced toxicity, more excellent stability, enhanced permeability and retention effect, and precise targeting. Nanoparticles are classified into several main categories. The nanoparticle drug delivery system is particular and utilizes tumor and tumor environment characteristics. Nanoparticles not only solve the limitations of conventional cancer treatment but also overcome multidrug resistance. Additionally, as new multidrug resistance mechanisms are unraveled and studied, nanoparticles are being investigated more vigorously. Various therapeutic implications of nanoformulations have created brand new perspectives for cancer treatment. However, most of the research is limited to in vivo and in vitro studies, and the number of approved nanodrugs has not much amplified over the years. This review discusses numerous types of nanoparticles, targeting mechanisms, and approved nanotherapeutics for oncological implications in cancer treatment. Further, we also summarize the current perspective, advantages, and challenges in clinical translation.

## Introduction

Cancer is a generic term for a set of diseases characterized by uncontrolled, random cell division and invasiveness. Extensive efforts over several years have been focused on detecting various risk factors for cancer. For some cancers, etiology has been influentially associated with specific environmental (acquired factors) such as radiation and pollution. However, an unhealthy lifestyle like a poorly balanced diet, tobacco consumption, smoking, stress, and lack of physical activity strongly impacts cancer risk determination [1, 2]. While these external factors have been recognized as major causes of cancer, the involvement of mutations of proto-oncogenes, tumor suppressor genes expression patterns, and the genes involved in DNA repair has been tough to estimate. Only 5–10% of cancer cases are linked with inherited genetics [3]. Advancing age is another crucial risk factor for cancer and many individual cancer types.

**Fig. 1:**
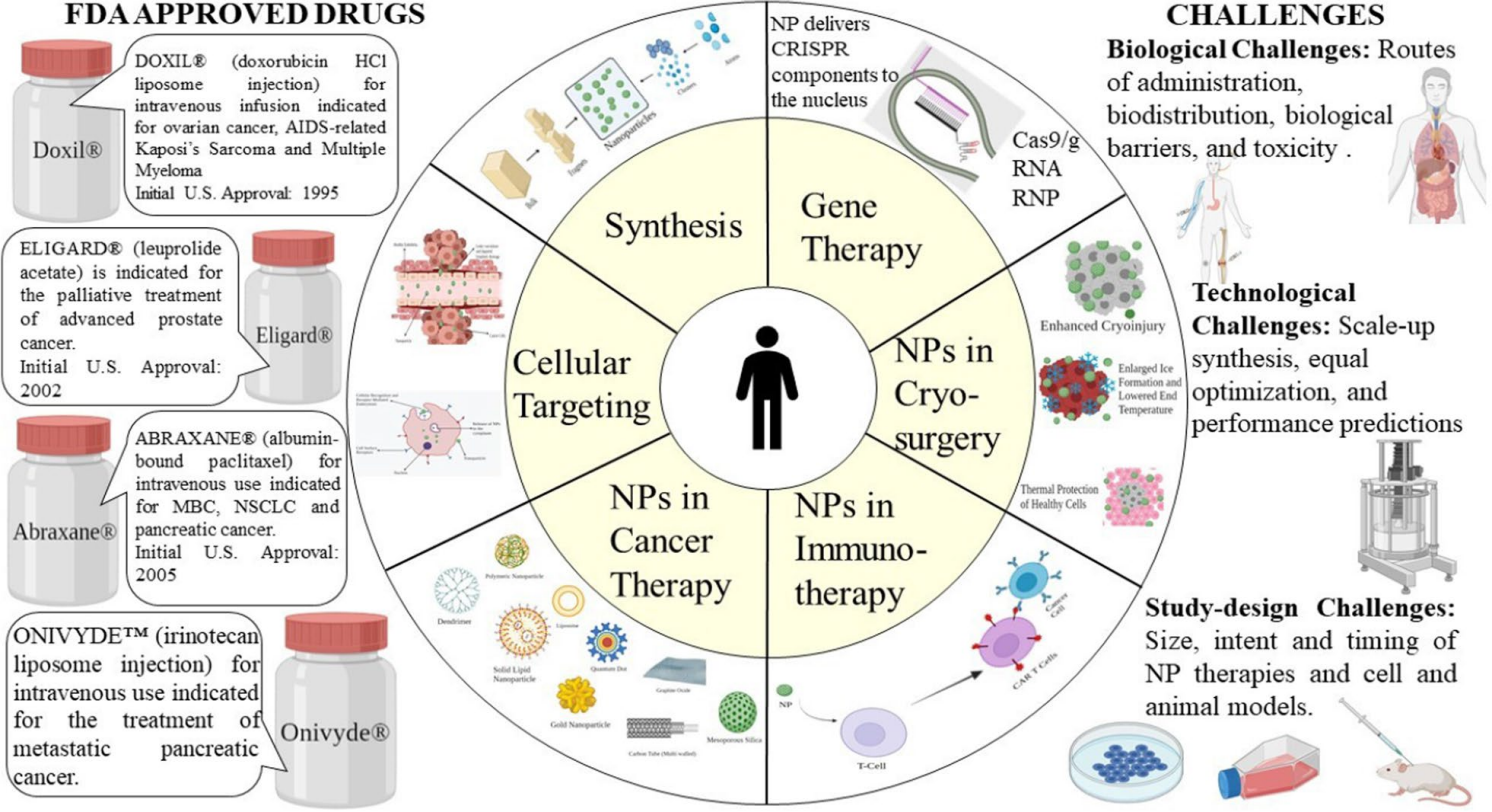
Nanoparticles for cancer therapy

Cancer is one of the significant public health problems globally and is the second leading cause of death. According to the American Cancer Society, the number of new cases is anticipated to be 1.9 million by the end of the year 2021 [4]. The conventional therapeutic approaches used in cancer treatment include surgery, chemotherapy, radiation therapy, targeted therapy, immunotherapy, and hormone therapy [5, 6]. Although chemotherapy and radiation therapy possess cytostasis and cytotoxicity abilities [7], these approaches are often linked with acute side effects and a high risk of recurrences. The most common side effects that are induced by include neuropathies, suppression of bone marrow, gastrointestinal and skin disorders, hair loss, and fatigue. Besides, there are a few drug-specific side effects such as anthracyclines and bleomycin-induced cardiotoxicity and pulmonary toxicity [8] (Fig. 1).

The advent of targeted therapy has made growth in precision therapy [9]. However, there are still many inevitable adverse effects, such as multi-drug resistance, limiting therapeutic efficacy [8]. Immunotherapeutic agents have yielded promising results by not only treating primary cancer but by preventing distant metastasis and lowering the rate of recurrence [10]. Nevertheless, autoimmune disease is a major side effect of immunotherapy. Additionally, studies and shreds of evidence suggest that immunotherapy is less effective against solid tumors than lymphoma [11]. These cancers create an unusual extracellular matrix (ECM) which is quite challenging for immune cells to infiltrate [12]. These newly evolved targeted therapies and immunotherapies interfere with signaling pathways that are vital in malignant behaviors and normal homeostatic functions of the epidermis and dermis and cause dermatologic adverse events (dAEs) [13].

Considering all of these details, the demand for the advancement of novel strategies for seeking precise therapy of cancer has gained momentum in recent years. Recent efforts have been made to address the limitations of existing therapeutic approaches using nanoparticles. Nanoparticle-based drug delivery systems have reflected benefits in cancer treatment and management by demonstrating good pharmacokinetics, precise targeting, reduced side effects, and drug resistance [14, 15].

On the heels of the advancements of nanotechnology, a number of nanotherapeutic drugs have been commercialized and are widely marketed, and many more have entered the clinical stage since 2010. Nanotherapeutic drugs have made progress in the domain of drug delivery systems and anti-tumor multidrug resistance (MDR) by providing a chance for drug combination therapy and inhibition of drug resistance mechanisms [16]. The pioneer effort was made to apply nanotechnology in medicine at ETH Zurich in the 1960s [17]. This combination has proved to be a better amalgamation in developing various diagnostic devices and better therapies. This review mainly focuses on basic principles of the application of nanotherapeutics, current challenges prospects, and describes the path of future research.

## Nanoparticles

Nanoparticles (NPs) are technically defined as particles with one dimension less than 100 nm with unique properties usually not found in bulk samples of the same material [18]. Depending on the nanoparticle’s overall shape, these can be classified as 0D, 1D, 2D or 3D [19]. The basic composition of nanoparticles is quite complex, comprising the surface layer, the shell layer, and the core, which is fundamentally the central portion of the NP and is usually termed as the NP itself [20]. Owing to their exceptional features like high surface: volume ratio, dissimilarity, sub-micron size, and enhanced targeting system, these materials have gained a lot of importance in multidisciplinary fields.

NPs are found to have deep tissue penetration to increase enhanced permeability and retention (EPR) effect. Besides, the surface characteristics impact bioavailability and half-life by effectively crossing epithelial fenestration [21]. For example, NPs coated with polyethylene glycol (PEG), a hydrophilic polymer, decrease opsonization and circumvent immune system clearance [22]. Additionally, it is possible to optimize the release rate of drugs or active moiety by manipulating particle polymer characteristics. Altogether, the distinct properties of NPs regulate their therapeutic effect in cancer management and treatment.

### Synthesis of NPs

**Fig. 2:**
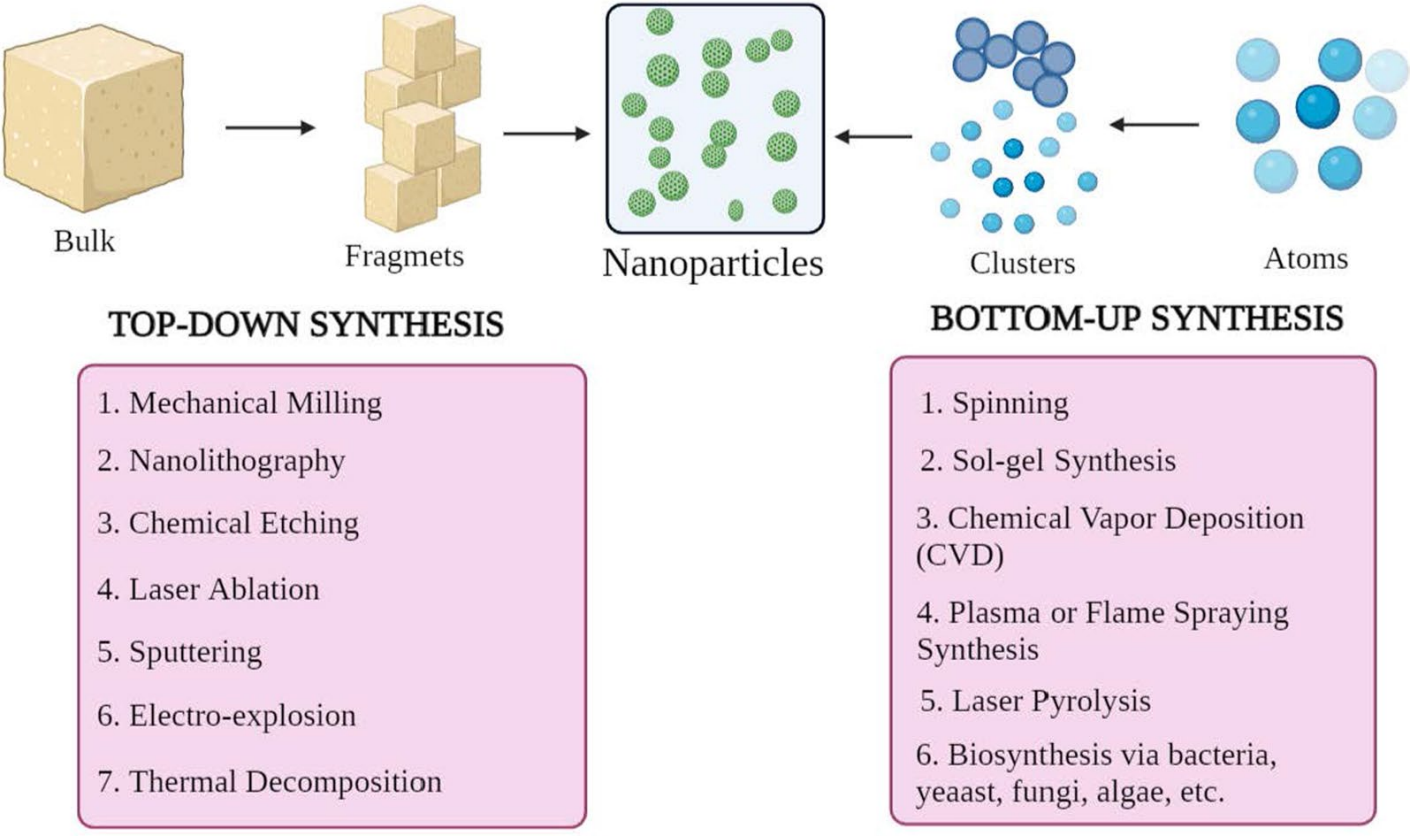
Classification of NP synthesis **a** top-down and **b** bottom-up approaches

The NPs are of different shapes, sizes, and structures. To achieve this, numerous synthesis methods are adopted. These methods can be largely categorized into two major groups: 1) bottom-up approach and 2) top-down approach. These approaches can be further classified into different subclasses based on reaction conditions and operation (Fig. 2).

#### Bottom-up Approach

This method involves building material from atoms to clusters to NPs, i.e., building from simpler substances, hence known as constructive method [23]. Some commonly used methods are spinning, solgel synthesis, chemical vapor deposition (CVD), plasma or flame spraying synthesis, laser pyrolysis, and biosynthesis.

#### Top-Down Approach

It is also known as the destructive method, which reduces bulk material or substance to synthesize NPs. A larger molecule is broken down or decomposed into smaller units that are converted into NPs [24]. It includes techniques such as mechanical milling, nanolithography, chemical etching, laser ablation, sputtering, electro-explosion, and thermal decomposition.

Remarkably, the morphological parameters such as size, shape and charge of NPs can be modified by changing the reaction conditions and other synthesis parameters [25]. Besides, the growth mechanism also determines the chemical properties of NPs. Hence understanding the growth mechanism is essential to synthesize required NPs.

## Mechanisms of Cellular Targeting

For effective cancer therapy, it is essential to develop or engineer a drug or gene delivery system that has an excellent ability to target tumor cells sparing the normal healthy cells. It enhances therapeutic efficacy, thereby shielding normal cells from the effect of cytotoxicity. It can be achieved by the well-organized delivery of NPs into the tumor microenvironment (TME), indirectly targeting cancer cells. These nanoformulations should pass through numerous physiological and biological barriers. These barriers are complex systems of several layers (epithelium, endothelium, and cellular membranes) and components (mechanical and physicochemical barriers and enzymatic barriers). These facts impose specifications with respect to the size, biocompatibility, and surface chemistry of NPs to prevent unspecific targeting. However, mere cytosolic internalization of an NP drug molecule does not mean it reaches its subcellular target. Specific engineering and optimization are mandatory to enable cellular or nuclear targeting.

Several studies have been carried out so far and several more are in progress to discover NP-based drug targeting design. These nanocarriers typically should possess certain fundamental characteristics such as 1) ability to remain stable in the vascular system (blood) until they reach their target, TME, 2) to escape the reticuloendothelial system (RES) clearance, 3) escape mononuclear phagocyte system (MPS), 4) accumulate in TME via tumor vasculature, 5) high-pressure penetration into the tumor fluid, and 6) reach the target and only interact with tumor cells [26]. The vital aspects such as surface functionalization, physicochemical properties, and pathophysiological characteristics regulate the process of NP drug targeting.

Generally, NPs considered apt for cancer treatment have a diameter range of 10–100 nm. In order to understand the process of interaction and crosstalk between NP carriers and cancer cells and tumor biology, it is important to address the targeting mechanisms. The targeting mechanisms can be broadly classified into two groups, passive targeting and active targeting.

### Passive Targeting

The observation of preferential accumulation of few macromolecules in cancer cells was found in the late 1980s. The first macromolecule to be reported to accumulate in the tumor was poly(styrene-co-maleic acid)-neocarzinostatin (SMANCS) by Matsuura and Maeda [27]. On further studies, this preferential distribution was attributed to the occurrence of fenestrations that are found in the damaged tumor blood vessels and to the poor lymphatic drainage, the amalgamation of which is known as “enhanced permeation and retention effect.”

Under certain conditions such as hypoxia or inflammation, the endothelium layer of the blood vessels becomes more permeable [28]. Under hypoxia situations, the rapidly growing tumor cells tend to put in action more blood vessels or engulf the existing ones to cope up. This process is known as neovascularization. These new blood vessels are leaky as they have large pores that lead to poor perm-selectivity of tumor blood vessels compared to the normal blood vessels [29, 30]. These large pores or fenestrations range from 200 to 2000 nm depending on the cancer type, TME and localization [31]. This rapid and defective angiogenesis provides very little resistance to extravasation and permits NPs to diffuse from such blood vessels and ultimately collect within cancer cells.

In normal tissues, the drainage of ECF (extracellular fluid) into lymphatic vessels frequently happens at an average flow velocity of 0.1–2 µm/s, which maintains constant drainage and renewal [32]. When a tumor is formed, the lymphatic function gets derailed, which results in minimal interstitial fluid uptake [33]. This feature contributes to the NPs retention as they are not cleared and hoard in the tumor interstitium. This process denotes the enhanced retention part of the EPR effect. This exceptional feature does not apply to molecules with short circulation time and gets washed out rapidly from the cancer cells. Hence, to improve such situations, encapsulating these small molecules in nanosized drug carriers is routinely carried out to enhance their pharmacokinetics, provide tumor selectivity and reduce side effects [34].

Over the EPR effect, TME is a vital feature in passive targeting. One of the important metabolic features of rapidly proliferating tumor cells is glycolysis. It is the chief energy source for cell division [35] and makes the surrounding environment acidic. This lowered pH of TME can be exploited to use pH-sensitive NPs that release drugs at low pH [36].

**Fig. 3:**
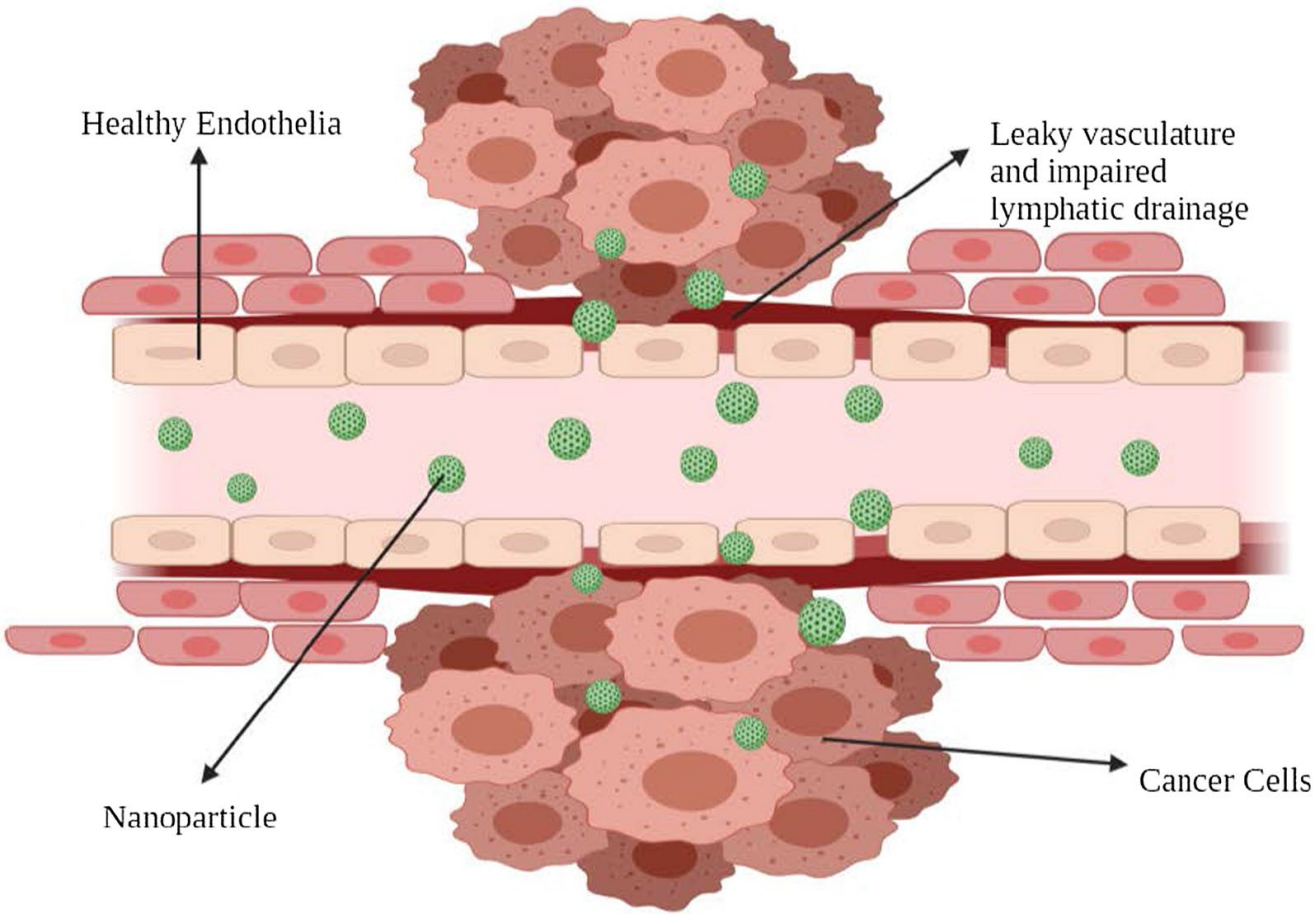
Passive cellular targeting

This type of tumor-targeting is termed as “passive.” Passive targeting mainly relies on different tumor biology (vascularity, leakiness) and carrier characteristics (size and circulation time). This type of tumor-targeting does not possess a specific ligand for certain types of tumor cells. The EPR effect greatly relies on the fundamental tumor biology, such as 1) the degree or extent of angiogenesis and lymphangiogenesis, 2) the extent or degree of perivascular tumor invasion, and 3) intratumor pressure. These factors, combined with physicochemical characteristics of NPs, determine the efficiency of NP drug delivery system (Fig. 3).

#### Examples of Passive Targeting

Taxanes are one of the most successful drug groups that are used in cancer treatment. Paclitaxel has shown great potency against a broad range of cancers. Breast cancer, lung cancer (small cell and non-small cell), and ovarian cancer are the most treated histologies with taxanes. US-FDA, in 2005, approved Abraxane® (albumin-bound paclitaxel, Abraxis Bio-Sciences), which is used for advanced or metastatic breast cancer (MBC).

Abraxane® is an anti-microtubule drug that stabilizes the microtubules by preventing depolymerization. It occurs when the drug encourages the microtubule assembly from tubulin dimers. This gained stability hinders microtubules reorganization, which is very important during interphase and mitotic cellular functions. During cell cycle and mitosis, paclitaxel, a well-used taxane, triggers unusual microtubules array along with multiple asters, respectively. Abraxane® alone or combined with another cytotoxic agent such as gemcitabine diminishes pancreatic stroma in pancreatic cancer xenograft mouse models [37].

Genexol PM® is an innovative nanoformulation of paclitaxel and sterile lyophilized polymeric micellar formulation without CrEL. Genexol PM®, according to trials, was found to have a three-times higher maximum tolerated dose (MTD) in nude mice. Besides, the biodistribution exhibited two- to three-times higher levels in different tissues such as liver, spleen, kidney, and lung and more prominently in cancer cells. It has been approved in South Korea to treat MBC. It is still under phase II clinical study in the USA to treat pancreatic cancer [38].

DaunoXome® (liposomal daunorubicin; Gilead Science/Diatos) is an anticancer medicine that reduces tumor cell growth. The active substance is daunorubicin. It is a unique formulation of daunorubicin (in liposome form) used to treat Kaposi’s sarcoma, a form of cancer that affects the skin, lungs, and intestines. US-FDA approved this in 1996 [39].

Although neovascularization and angiogenesis influence NP diffusion, it leads to greater interstitial pressure, which inhibits the accumulation of NPs. Moreover, due to heterogenous blood supply, the growth of the tumor cells is irregular, i.e., the cells that are close to blood vessels divide faster than those that are away from the blood vessel or deep in the core-forming hypoxic or necrotic area within the tumor. This irregular leaking, which causes high interstitial pressure, impedes drug delivery and accumulation and slows down the neovascularization process [34]. However, it is possible to control EPR effect, either mechanically or chemically. These include nitric oxide, peroxynitrate, bradykinin, VPF (vascular permeability factor), ultrasound, radiation, hyperthermia, etc. However, there are certain limitations and contra-indications.

### Active Targeting

Active targeting depends on specific ligands or molecules, like transferrin and folate, which binds to molecules or receptors that are specifically expressed or over-expressed on the target cells (diseased organs, tissues, cells or subcellular domains) [40]. This type of targeting is called ligand-mediated targeting [41]. Here, the NPs that possess ligand with specific functions such as retention and uptake need to be in the target's proximity so that there is greater affinity. This strategy enhances the changes of NPs binding to the cancer cell, enhancing the drug penetration. The foremost indication of the same was observed in 1980 with antibodies grafted in the surface of liposomes [34], followed by other various types of ligands like peptides, aptamers. Hence, the main method is intended at increasing the crosstalk between NPs and the target without fluctuating the total biodistribution [42]. The vital mechanism of active targeting or ligand-mediated targeting is ligand identification by the target substrate receptors. The illustrative ligands may include proteins, peptides, antibodies, nucleic acids, sugars, small molecules like vitamins, etc. [43]. The most commonly studied receptors are transferrin receptor, folate receptor, glycoproteins and the epidermal growth factor receptor (EGFR). Ligand-target interaction triggers infolding of the membrane and internalization of NPs via receptors-mediated endocytosis. There are various mechanisms by which active targeting takes place. The majority of tumor-targeting is done by the tumor cell targeting in general by NPs. This process improves cell penetration. As stated before, transferrin is one of the widely studied receptors. It is a type of serum glycoprotein that aids in transporting iron into cells. These receptors are found to be overexpressed in most tumor cells, especially solid tumors and are expressed at lower levels in healthy cells. Hence, we can modify the NPs with associated ligands that specifically target transferrin [44]. For instance, A2780 ovarian carcinoma cells overexpress transferrin. This feature is used by transferrin-modified PEG-phosphatidyl-ethanolamine (Tf-Mpeg-pe) NPs that specifically target such cells [45]. Another alternative method is to target cells adjacent to cancer cells, such as angiogenic endothelial cells. These cells also have close contact with tumor blood vessels. This strategy makes it possible to create hypoxia and necrosis by reducing the blood supply to the cancer cells. It has been found out that tumor tissues are more acidic than normal ones. This has been extensively explained by the Warburg effect [46]. This explains the shift of cancer cell metabolism into glycolysis, forming lactic acid. When the lactic acid accumulates, the cell dies. To cope with this situation, the cells start overexpressing proton pumps that pump out excess lactic acid into the extracellular environment, making it more acidic. Therefore, liposome-based pH-sensitive drug delivery system has been studied.

**Fig. 4:**
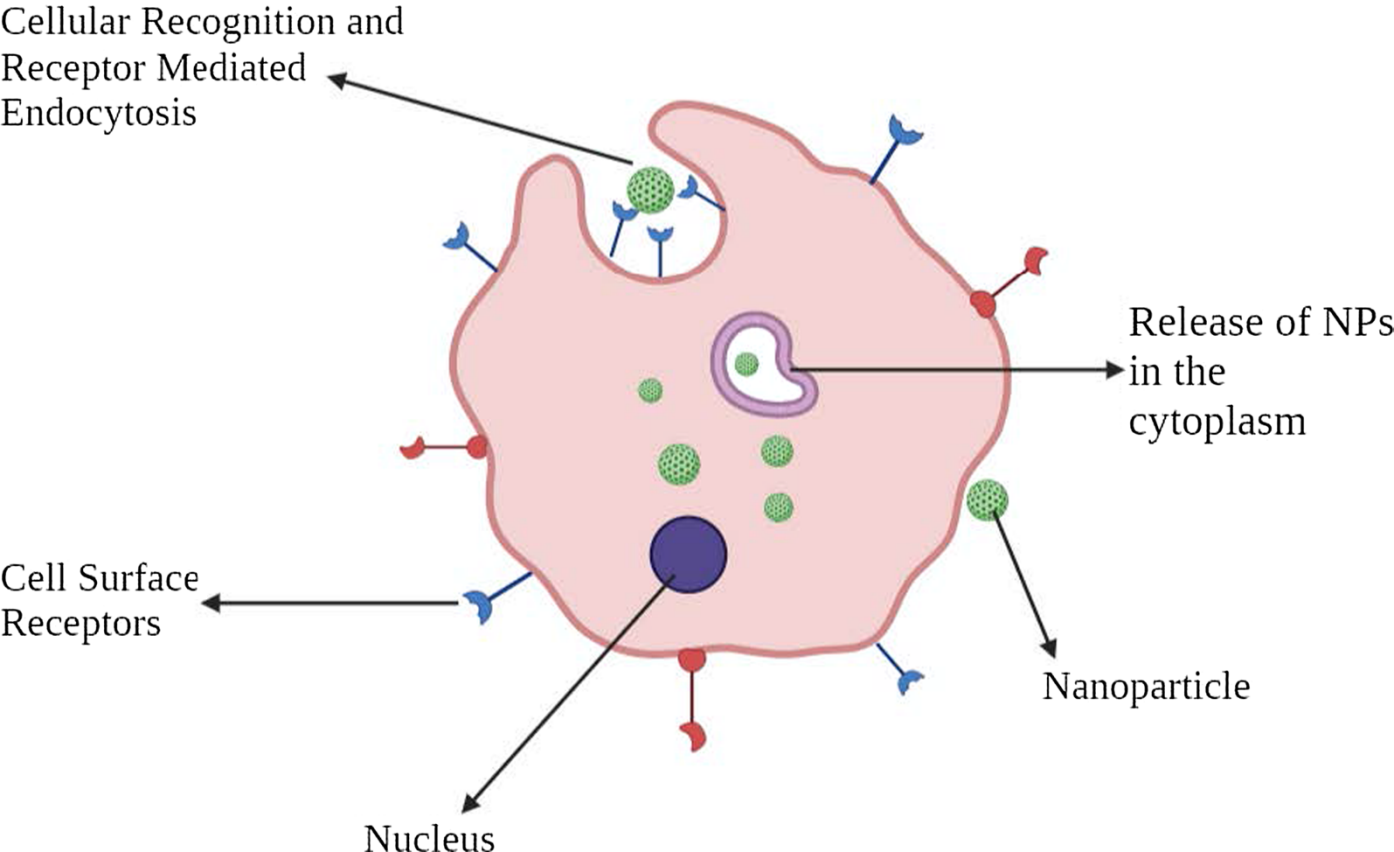
Pictorial representation of active cellular targeting

The multivalent nature of the NPs improves the crosstalk of ligand coated NPs with target cancer cells. The design of such NPs is complex as NP architecture and ligand-target chemistry influence the efficacy of the entire method. Other factors such as route of administration, physicochemical properties such as ligand density [47], and size of NPs [8] contribute to the system's success (Fig. 4).

#### Examples of Active Targeting

EGFR, a member of the ErbB family of tyrosine kinase (TK) receptors, is overexpressed in various types of cancer, especially with squamous cell histology. Gold NPs with anti-EGFR-PEG-AuNPs and anti-IgG-PEG-Au nanoparticles can be used to target the human SCC [48].

Herceptin® is a therapeutic drug that targets human EGF receptor-2 (HER2) that is overexpressed on breast cancer cell surfaces. HER2-targeted PEGylated liposomal doxorubicin was developed to reduce cardiotoxicity, a known side effect of anthracyclines [49].

The surface of the tumor endothelium expresses a glycoprotein known as vascular cell adhesion molecule-1 (VCAM-1) that is involved in the process of angiogenesis. A study has highlighted NPs that target VCAM-1 in the breast cancer model, indicating its potential role [50].

Folic acid, also known as vitamin B9, is vital in nucleotide synthesis. Folic acid is internalized by the folate receptor that is expressed on the cells. However, tumor cells overexpress FR-α (alpha isoform of folate receptor), while FR-β is overexpressed in liquid cancer cells [51]. Targeting the folate receptors by NPs has been currently for specific cancer treatments [52, 53].

## Nanoparticles in Cancer Therapy

**Fig. 5:**
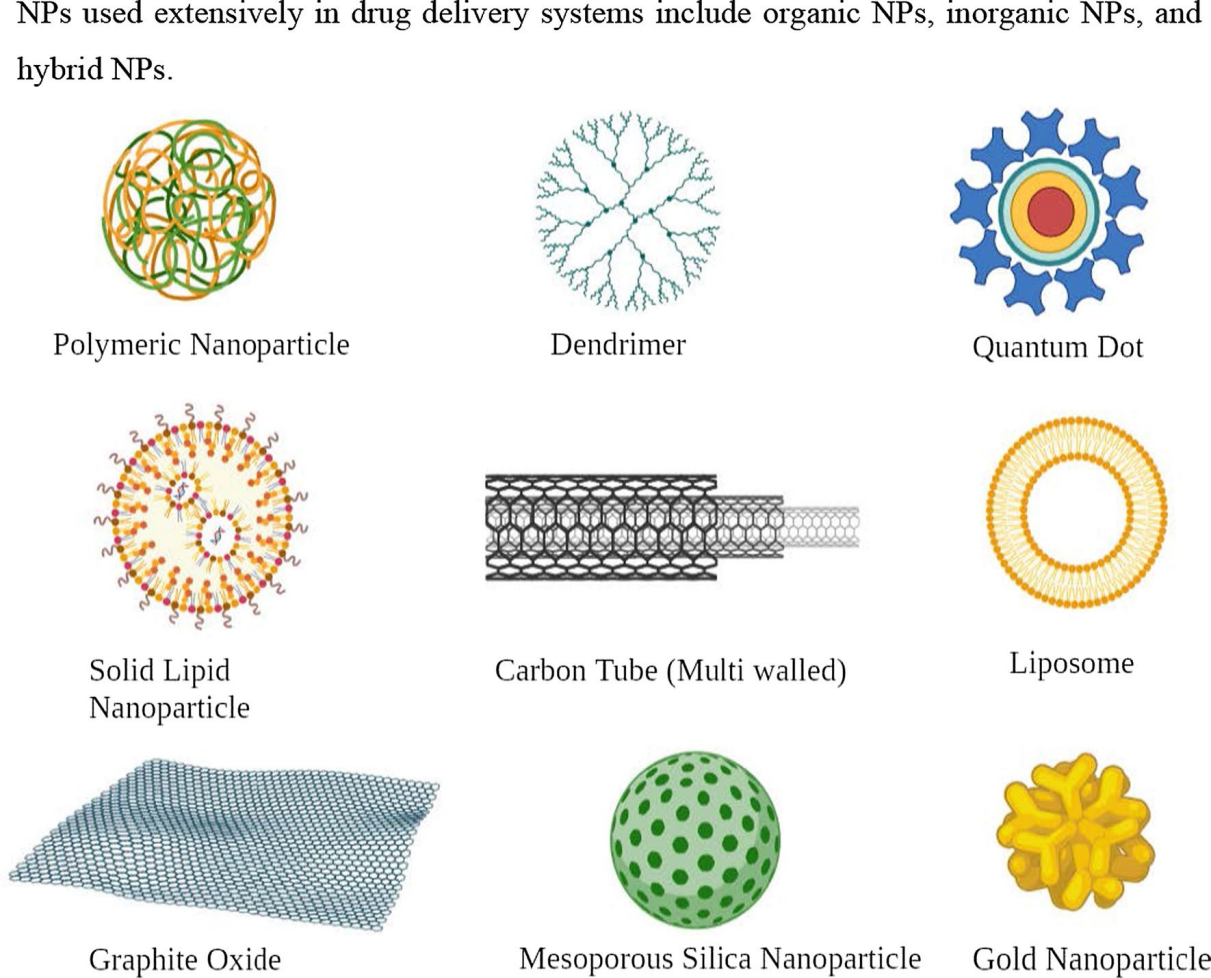
Various types of nanomaterials used in cancer therapy

NPs used extensively in drug delivery systems include organic NPs, inorganic NPs, and hybrid NPs (Fig. 5).

### Organic Nanoparticles

#### Polymeric Nanoparticles

Polymeric nanoparticles (PNPs) are well-defined as “colloidal macromolecules” with specific structural architecture formed by different monomers [54]. The drug is either entrapped or attached to NPs’exterior, creating a nanosphere or a nanocapsule to achieve regulated drug release in the target [55]. Initially, PNPs were made up of non-biodegradable polymers such as polyacrylamide, polymethylmethacrylate (PMMA), and polystyrene [56]. However, the accumulation of these led to toxicity due to difficulty in eliminating these from the system. Biodegradable polymers such as polylactic acid, poly(amino acids), chitosan, alginate, and albumin are now being used and are known to reduce toxicity and enhance drug release and biocompatibility [57]. Proven research has reflected that by coating PNPs with polysorbates and by using polysorbates surfactant effect. Exterior coating enhances NPs' interactions with the endothelial cell membrane of the blood–brain barrier (BBB) [58].

A study showed that nanocapsules loaded with indomethacin involved a substantial decrease in the size of the tumor and improved survival in a xenograft glioma model in rats [59]. This is a growing field with more than ten polymeric NPs containing anticancer drugs are under clinical development. A few examples include paclitaxel poliglumex (Xyotax), PEG-camptothecin (Prothecan), Modified dextran-camptothecin (DE 310), HPMA copolymer-DACH-platinate (AP5346), HPMA copolymer-platinate (AP 5280), HPMA copolymer-paclitaxel (PNU166945), and HPMA copolymer-doxorubicin galactosamine (PK2) [60].

#### Dendrimers

Dendrimers are spherical polymeric macromolecules with defined hyperbranched architecture. Highly branched structures are the characteristic feature of dendrimers. Typically, the synthesis of dendrimers is initiated by reacting an ammonia core with acrylic acid. This reaction results in forming a “tri-acid” molecule that further reacts with ethylenediamine to yield “tri-amine,” a GO product. This product further reacts with acrylic acid to give rise to hexa-acid, which further produces “hexa-amine” (Generation 1) product and so on [61]. Typically, the size of the dendrimers ranges from 1–10 nm. However, the size may reach up to 15 nm [62]. Given their specific structure like defined molecular weight, adjustable branches, bioavailability, and charge, these are used to target nucleic acids. Some dendrimers that are widely used are polyamidoamine (PAMAM), PEG (poly(ethyleneglycol)), PPI (polypropylenimine), and TEA (triethanolamine) [63].

A PAMAM dendrimer was initially designed to achieve MDR management. DNA assembled PAMAM dendrimers have been described extensively. As compared with animals treated with single-agent chemotherapy, the synthesized dendrimers significantly delayed the growth of epithelial cancer xenografts [64].

#### mAb Nanoparticles

Monoclonal antibodies are widely used in cancer treatment for their particular targeting abilities [65]. These mAb are now combined with NPs to form antibody–drug conjugates (ADCs). These are proved to be highly specific and compelling than cytotoxic drugs or mAb alone. For instance, an antibody–drug NP consisting of paclitaxel core and a surface modified with trastuzumab presented a better anti-tumor efficacy and lower toxicity than single-agent paclitaxel or trastuzumab alone in HER2 positive breast epithelial cell control [66].

#### Extracellular Vesicles

Extracellular vesicles (EVs) are double-layered phosphor-lipid vesicles ranging from 50–1000 nm n size [67]. EVs are continuously secreted by different cells types and vary in origin, size, and composition. EVs are divided into three classes: 1) exosomes, 2) microvesicles, and 3) apoptotic bodies [68]. NPs combined with exosomes are widely used as they have lipid and molecules that are very similar to origin cells. Besides, they escape the immune surveillance and internalize very quickly within the cancer cells. They act as natural vehicles by delivering cytotoxic drugs and other anti-tumor drugs to the target sites. Exosomes loaded with doxorubicin (exoDOX) are the best example. exoDOX is used to treat breast cancer and has shown great results compared to conservative treatment with doxorubicin by enhancing the cytotoxicity and avoiding cardiotoxicity [69]. Exosome NPs have intrinsic biocompatibility features, advanced chemical stability, and intracellular communications compared to synthetic NPs. Nevertheless, drawbacks like deficiency of standard conditions for exosomal isolation and purification are crucial and need to be addressed [70, 71].

#### Liposomes

These are spherical vesicles comprising phospholipids that may be either uni-lamellar or multi-lamellar to encapsulate drug molecules [72]. Liposomes are unique in having characteristics such as low intrinsic toxicity, weak immunogenicity, and biological inertness [73]. Liposomes are the first nanoscale drug that was approved in 1965 [74]. A typical liposome structure is composed of a “hydrophilic core” and a “hydrophobic phospholipid bilayer.” This unique architecture makes it possible for them to entrap both hydrophilic and hydrophobic drugs to effectively protect the entrapped drug from environmental degradation in circulation [75].

Liposomes provide an excellent platform for drug delivery such as doxorubicin, paclitaxel, and nucleic acid as well by demonstrating higher anti-tumor efficacy and enhanced bioavailability [76]. Doxil® and Myocet® are approved liposome-based formulations of daunorubicin used to treat MBC [77, 78]. However, due to shortcomings like decreased encapsulation efficacy, speedy removal by MP, cell adsorption, and short shelf life, the application of liposome-based NPs is limited.

#### Solid Lipid Nanoparticles (SLN)

They are colloidal nanocarriers (1–100 nm) made up of a phospholipid monolayer, emulsifier, and water [79]. These are known as zero-dimensional nanomaterials. The lipid component may be triglycerides, fatty acids, waxes, steroids, and PEGylated lipids [80]. Unlike conventional liposomes, SLNs have a “micelle-like structure” within which the drug is entrapped in a non-aqueous core. Examples include mitoxantrone-loaded SLN, which has shown reduced toxicity and enhanced bioavailability [81]. The incorporation of doxorubicin and idarubicin by SLN in “P388/ADR leukemia cells” and the “murine leukemia mouse model” has shown positive results [82].

#### Nanoemulsions

Nanoemulsions are colloidal NPs with heterogeneous mixtures of an oil droplet in aqueous media ranging from 10–1000 nm [83]. Three representative types of nanoemulsions can be made in: 1) oil-in-water system, 2) water-in-oil system, and 3) bi-continuous nanoemulsions. Membrane-modified nanoemulsions have been extensively studied. For instance, nanoemulsions loaded with spirulina and paclitaxel showed an improved anti-tumor effect by regulating immunity through TLR4/NF-kB signaling pathways [84]. Nanoemulsion consisting of rapamycin, bevacizumab, and temozolomide is known to treat advanced melanoma [85]. Nanoemulsions are different from liposomes and certainly have enhanced characteristics than others, such as optical clarity, stability, and biodegradability [86]. However, there are challenges to clinical applications of these nanoemulsions as these involve high temperature and pressure and instruments such as homogenizers and microfluidizers that are expensive.

#### Cyclodextrin Nanosponges

Cyclodextrins are usually used as stabilizers to increase the drug loading capacity of NPs [87]. Nanosponges are tiny, mesh-like structures [88]. Β-cyclodextrin nanosponges loaded with paclitaxel have shown sound cytotoxic effects in MCF-7 cell line culture [89]. Similarly, camptothecin has shown improved solubility and stability when formulated with cyclodextrin-based nanosponges [90].

### Inorganic Nanoparticles

#### Carbon Nanoparticles

Carbon NPs as the name suggests are based on the element carbon. They have been widely utilized in medical arenas because of their optical, mechanical, and electronic properties combined with biocompatibility [91]. Due to their inherent hydrophobic nature, carbon NPs can encapsulate drugs through π-π stacking [92]. Carbon NPs are further categorized into graphene, carbon nanotubes, fullerenes, carbon nanohorns, and graphyne. Although all these are carbon-based, they vary in their structure, morphology, and properties.

“Graphene” is 2D crystal with sp2-hybridized carbon sheet that holds extraordinary mechanical, electrochemical, and high drug loading properties. Further, based on composition, properties, and composition, graphene can be divided as follows: 1) single-layer graphene, 2) graphene oxide (GO), 3) reduced graphene oxide (rGO), and 4) multi-layer graphene [93]. GO and rGOs are widely used due to their ability to target hypoxia [94] and irregular angiogenesis in TME [95]. Studies have shown that GO-doxorubicin exhibits higher anticancer activities in cellular models of breast cancer [96].

Fullerenes are large carbon-cage molecules composed of carbon allotrope with different conformation types such as sphere, ellipsoid, or tube. They are the most widely studied nanocarriers as they have typical structural, physical, chemical, and electrical properties [97]. These are used in photodynamic therapy as they have triple yield and generate oxygen species due to the presence of extended π-conjugation and the ability to absorb light [98]. PEG-modified fullerenes showed promising photodynamic effects on tumor cells [99].

Carbon nanotubes (CNTs) are cylindrical tubes, most often considered as rolls of graphene, were discovered in the late 1980s. They are classified into two groups: 1) single-walled CNTs and 2) multi-walled CNTs. As they are carbon-based, they can bring upon immune response by interacting with immune cells, thereby suppressing the tumor growth. Traditionally, they have been used as DNA delivery vectors and for thermal ablation therapy. For instance, a fluorescent single-walled CNT with mAb encapsulating doxorubicin is used to target colon cancer cells. Such CNTs form a complex which is effectively engulfed by the cancer cells leading to the intracellular release of doxorubicin, whereas the CNTs are retained in the cytoplasm [100].

#### Quantum Dots

Quantum dots are typically nanometer-scale semiconductors with a broad spectrum of absorption, narrow emission bands, and high photostability, allowing them to be widely used in biological imaging [101]. Based on carbon, these are divided into: 1) graphene quantum dots, 2) nanodiamond quantum dots, and 3) carbon quantum dots. Besides biological imaging, quantum dots are being actively investigated in cancer treatment. The most commonly used quantum dots is graphene quantum dots due to their inherent biocompatibility and rapid excretion. For example, quantum dots aptamer—doxorubicin conjugate targets prostate cancer cells [102]. However, the deficiency of optimized process in producing quantum dots is the major obstacle.

#### Metallic Nanoparticles

Metallic nanoparticles are commonly explored in “biological imaging” and targeted DDS due to their remarkable optical, magnetic, and photothermal properties. Some of the most commonly used metallic NPs are gold NPs, silver NPs, iron-based NPs, and copper NPs. Gold NPs are used as intracellular targeting drug carriers because the size and surface properties are easily controlled [103]. Moreover, their visible light extinction behavior makes it possible to track NP trajectories in the cells. “Anti-HER2 functionalized gold-on-silica nanoshells” have been shown to aim HER2 positive breast cancer cells [104]. Combidex®, an iron oxide NP formulation, is presently in the late-stage clinical testing phase to detect nodal metastases [105]. Feraheme®, a ferumoxytol containing iron oxide NP formulation, is used to treat iron-deficiency anemia. This is also used to treat nodal metastases in prostate and testicular cancer and was approved by FDA in June 2009 [106, 107].

#### Magnetic Nanoparticles

Magnetic NPs are generally used in MRI imaging, and drug delivery contains metal or metal oxides. These are usually covered with organic substances like polymers and fatty acids to enhance stability and biocompatibility [108]. LHRH-conjugated superparamagnetic iron oxide NPs are effective in targeting and imaging of breast cancer [109]. Moreover, magnetic NPs are used in magnetic hyperthermia for thermal ablation of cancer cells [110, 111]. Some of the magnetic NPs that are in the market or in the clinical trial phase are Feridex® and Resovist® for liver metastasis and colon cancer [112].

#### Calcium Phosphate Nanoparticles

“Calcium phosphate NPs” is biologically compatible, biodegradable, and do not cause any harsh adverse reactions. Hence, they are used as a delivery agent for insulin, growth factors, antibiotics, and contraceptives [113]. They are also used in the delivery of oligonucleotides and plasmid DNA [114]. Calcium phosphate NPs combined with either viral or non-viral vector has been positively used as delivery vectors in cellular gene transfer. A “liposomal nanolipoplex formulation” of calcium and glycerol has shown decreased toxicity and enhanced transfection features [115, 116].

#### Silica Nanoparticles

Silica being a significant component of many natural materials was only studied concerning biology recently. Silica NPs are commonly used to deliver genes by functionalizing the NP surface with amino-silicanes [117]. N-(6–aminohexyl)–3–aminopropyl–trimethoxysilane functionalized silica NPs have shown excellent efficiency in the transfection of Cos-1 cells with minimal toxicity and is now commercially available [118]. Mesoporous silica NPs are considered one of the best drug carriers due to their better pharmacokinetic properties. They have been extensively used in immunotherapy. According to a study, colorectal cancer cells have shown successful uptake of camptothecin-loaded mesoporous silica NPs.

## Mechanism of NPs in Overcoming Drug Resistance

Drug resistance is one of the chief problems in cancer therapy and management. It prevails across all types of cancer and all possible treatment modalities. Drug resistance is a phenomenon that results when diseases become tolerant to pharmaceutical treatments. Drug resistance can be classified into two types: 1) innate and 2) acquired [119]. Innate resistance usually results from pre-existing mutations in the genes that are involved in cell growth or apoptosis. Acquired resistance is defined as the type of resistance that is developed after a particular anti-tumor treatment, which may result from the development of new mutations or from alterations in the TME during treatment. Nanoparticles, due to their extraordinary ability to co-encapsulate multiple therapeutic agents, can also be used to overcome cancer-related drug resistance.

### Targeting Efflux Transporters

Efflux transporters are classified under the family of “ATP-binding cassette (ABC) transporters.” These have a significant role in MDR. The primary function of these transporters is to pump out drugs out of the cell and reduce the concentration. “P-glycoprotein (P-gp)” is one such efflux transporter that is overexpressed by drug-resistant cancer cells [120, 121].

Overexpression of P-gp has been linked with inadequate treatment response, especially in breast cancer [122] and ovarian cancer [123]. NPs can be used to tackle efflux pumps. As NPs internalize the cell via “endocytosis” instead of diffusion and release the drug at the “perinuclear site,” which is distant from active efflux pumps, NPs can bypass the efflux pumps [124]. Besides, by modifying the control of drug releases, such as by utilizing low pH levels and redox as triggers, NPs can effectively bypass efflux pumps [125, 126].

Combination therapy is yet another method to overcome MDR. NPs can be loaded with multiple drugs within a single drug carrier [127]. Inhibiting efflux transporter expression instead of just dodging them would be another viable option. This can be achieved by building NPs in such a way that it can entrap both efflux pump inhibitors and chemotherapy agents [128]. A recent study positively reflected upon reversing MDR in breast cancer cells by using NPs that co-deliver COX-2 inhibitors and doxorubicin [129]. Similarly, using silica NP that encapsulates miRNA-495 and doxorubicin has proved effective in overcoming drug resistance in lung cancer cells [130]. Another interesting study found out that using NPs in the tumor neo-vasculature targeting KDR receptors is a more effective anti-tumor function than P-gp inhibitor combination therapy. Yet, another way of overcoming drug resistance is by depleting the source of ATP, which is essential for the functioning of ABC transporters. This can be done by targeting mitochondria which leads to a decrease in ATP production.

### Targeting an Apoptotic Pathway

Cancer cells proliferate due to faulty apoptotic machinery and upsurge their survival adding to drug resistance [131]. The faulty apoptotic pathway gets activated by “deregulation of Bcl-2” and “nuclear factor kappa B (NF-κB).” These are the most widely investigated anti-apoptotic proteins and can be potentially used as the target for reversing drug resistance. Using a classic process of co-delivery of “Bcl-2 siRNA and chemotherapeutics” by NPs is a way to overcome MDR [132]. NF-κB inhibitors have been used in combination with “pyrrolidine dithiocarbamate (PDTC)” [133] and curcumin [134]. Besides suppressing anti-apoptotic factors, triggering pro-apoptotic factors is another to fight “apoptotic pathway-mediated drug resistance.” For instance, a combination of ceramide and paclitaxel is a good example [135]. Ceramide restores the expression of a chief tumor suppressor, p53 protein, by regulating alternative pre-mRNA splicing. Delivering ceramide via NPs is an excellent way to correct the p53 missense mutation [136]. Owing to its potential, a combination of ceramide and paclitaxel has shown significant therapeutic efficacy in cancer drug resistance models. Transfecting the p53 gene by cationic SLNs has been reported in lung cancer cases [137]. Similarly, transfecting the p53 gene by PLGA has been carried out in breast cancer cells models that have shown potent induction of apoptosis and inhibition of tumor growth [138].

Some NP-based DDS act by impeding efflux pumps and encouraging apoptosis [139]. A pioneering study conducted to prove both pump- and non-pump-mediated drug resistance used an “amphiphilic cationic NP” entrapping paclitaxel and Bcl-2 converter gene in drug-resistant liver cancer models. NP complex diminished P-gp-induced drug efflux and the apoptosis activation. Similarly, co-delivery of “doxorubicin and resveratrol encapsulated in NPs” has shown noteworthy cellular toxicity on doxorubicin resistance breast cancer cells by downregulating the expression of Bcl-2 and NF-κB, thereby initiating apoptosis as well as through the inhibition of efflux transporter expression [140]. A similar study was done on multi-drug resistant prostate cancer cells by using folic acid-conjugated planetary ball milled NPs encapsulated with resveratrol and docetaxel. This worked by downregulating anti-apoptotic gene expression while inhibiting ABC transporter markers [141].

### Targeting Hypoxia

Hypoxia is yet an additional aspect that backs MDR [142]. Due to abnormal blood vessels in the vicinity of the tumor and due to the increasing demand of oxygen by the rapidly growing tumor, some tumor cells are repeatedly in a hypoxic condition. The part of the tumor that is in hypoxic condition often escapes from the chemotherapy drugs. Hypoxia creates an oxygen ramp inside the tumor that intensifies tumor heterogeneity, encouraging a more aggressive phenotype. Moreover, the hypoxia condition has been established to facilitate the overexpression of efflux proteins [143]. The major protein, “hypoxia-inducible factor 1α (HIF-1α)” acts an important role. Hence targeting HIF-1α or silencing HIF-1α gene is a way to overcome drug resistance. NPs containing HIF-1α siRNA can be used to reduce hypoxia-mediated drug resistance [144]. Instead of directly targeting HIF-1α, indirect inhibition of HIF-1α signaling can be used. For example, the “PI3K/Akt/mTOR pathway” is known to control the expression of HIF-1α. Inhibition of this pathway effectively downregulates the expression of HIF-1α, which enhances the sensitivity of MDR cells to cancer treatment [145]. NPs like PLGA-PEG and PEGylated and non-PEGylated liposomes can be used effectively. In addition, “heat shock protein 90 (HSP90)” is needed for transcriptional activity of HIF-1 and inhibition of HSP90, which downregulates the expression of HIF-1α [146]. The HSP90 inhibitor in “17AAG loaded NPs” has dramatically improved MDR in bladder cancer treatment [147].

## Nanoparticles and Proteomics

When NPs are subjected to the biological system, they are surrounded by cellular and serum proteins which form a structure known as protein corona (PC) [148]. Based on the degree of interaction of these proteins with the NPs, there are classified into the hard corona and soft corona. “Hard corona” is formed when these proteins have a high binding affinity towards the NPs. “Soft corona” is produced when these proteins are loosely bound to the NPS. It has been established that the most protein forming a PC first will be eventually substituted by proteins with higher affinities. This is known as Vroman effect [149]. Hence developing the technology that can manufacture NPs with desired properties is essential. Several proteomic approaches such as MS, LC–MS, SDS-PAGE, isothermal microcalorimetry (ITC), etc. [150], are being used. PC affects the crosstalk of NP with the biological setting and thereby governs the application and usage of the same in the medical field.

Cancer proteomics studies the number of proteins in cancer cells and serum, which supports hunting proteins and biomarkers that aids in diagnosis, treatment, and prognosis [151]. It also helps in understanding cancer pathogenesis and drug resistance mechanism. Post-translational modifications (PTMs) play an indispensable part in occurrence, recurrence, and metastasis. Besides using chemotherapy and kinase inhibitors, novel agents like siRNA, mRNA, and gene editing are central therapeutics used with NPs.

### Nanotechnology for Small Interfering RNA (siRNA) Delivery

siRNAs are small ds RNA molecules (around 21 nucleotides long) that suppress the expression of genes in the target. This process is known as “RNA interference.” A few siRNA-based NPs that are currently under clinical investigations are ALN-TTR01 that is used to target the transthyretin gene to treat transthyretin-mediated amyloidosis, and Atu027, which is a liposomal siRNA that targets protein kinase N3 and TKM-ApoB that knock downs the expression of ApoB [152, 153].

### Nanotechnology for Tumor microRNA Profiling and Delivery

MicroRNAs are a class of endogenous “single-stranded non-coding RNA” molecules that control post-transcription gene expression by blocking translation of the target mRNA or repressing protein production by destabilizing mRNA [154]. These are emerging as vital biomarkers that are a significant target for cancer diagnosis, therapy, and treatment. The base priming nature of nucleic acid forms the very foundation for nanotechnology used miRNA profiling techniques. Several profiling techniques use biosensors or surface plasmon resonance imaging techniques in combination with molecular biology enzymatic reactions. Nanotechnology can be used for the delivery of MicroRNAs. For example, biodegradable polycationic prodrugs showed promising results in the regulation of polyamine metabolism [155]. MicroRNA-loaded polycation-hyaluronic acid NPs of single-chain antibody fragments have shown progressive downregulation of “survivin expression” in high metastatic cancer load in the lung of murine B16F10 melanoma.

### DNA Nanotechnology for Cancer Therapy

DNA-based nanostructures have been synthesized for DNA sensors to detect nucleic acid, DNA-coated gold NPs for lead sensing by hybridizing Pb-activated DNAzyme to the linking DNA, scaffolds to organize organics, inorganic, and biomolecules into distinct morphology molecular transporters, and drug delivery (Table 1).

**Table 1:** List of nanomedicines for cancer therapy approved by FDA [156–159]

Tradename	Material	Drug	Company	Indication	Year(s) approved
Doxil®	Liposome-PEG	Doxorubicin	Janssen	MBC, metastatic ovarian cancer	1995
Eligard®	PLGA	Leuprolide acetate	Tolmar	Prostate Cancer	2002
Abraxane®	Albumin	Paclitaxel	Celgene	Metastatic breast cancer	2005
Genexol PM®	mPEG-PLA	Paclitaxel	Samyang Corporation	Metastatic breast cancer	2007
Onivyde®	Liposome	Irinotecan	Merrimack	Pancreatic cancer	2015

## Advantages of Nanoparticles in Cancer Therapy

The utilization of nanotechnology in the diagnosis, treatment, and management of cancer has led to a whole new era. NPs, either by active or passive targeting, augment the intracellular concentration of drugs while avoiding toxicity in the healthy tissue. The targeted NPs can be designed and altered as either pH-sensitive or temperature-sensitive to establish and regulate the drug release. The pH-sensitive drug delivery system can deliver drugs within the acidic TME. Similarly, the temperature-sensitive NPs release the drugs in the target site due to changes in temperature brought in by sources like magnetic fields and ultrasound waves. In addition, the “physicochemical characteristics” of NPs, such as shape, size, molecular mass, and surface chemistry, have a significant part in the targeted drug delivery system. Further, NPs can be modified according to the target and used to target a particular moiety.

Conventional chemotherapy and radiation therapy have several disadvantages concerning efficacy and side effects because of uneven dispersal and cytotoxicity. Therefore, cautious dosing is required that effectively kills cancer cells without any significant toxicity. To reach the target site, the drug has to pass several fortifications. Drug metabolism is a very complex process. In physiological conditions, the drug needs to pass TME, RES, BBB, and kidney infiltration. RES or macrophage system is made up of “blood monocytes, macrophages, and other immune cells” [160]. MPS in the liver, spleen, or lungs react with the drugs and activate “macrophages or leukocytes” that rapidly remove the drug. This leads to a short half-life of the drug [161]. To overcome this, NPs with “surface modification,” such as PEG, bypass this mechanism and increase the “drug half-life.” Besides, kidney infiltration is a crucial function in the human body. Proper kidney infiltration thus minimizes the toxicity caused by NPs.

The brain-blood barrier (BBB) is a specialized protection structure offered to protect the CNS from harmful and toxic agents. “Brain capillary endothelial cells” are arranged in the form of a wall that provides essential nutrients to the brain. Since the primary function of BBB is to block toxic agents to reach the brain, currently available chemotherapy agents for brain cancer are highly limited to intraventricular or intracerebral infusions [162]. However, NPs are known to cross BBB. Now, several approaches such as EPR effect, focused ultrasound, peptide-modified endocytosis, and transcytosis are used to deliver NPs. Glutathione PEGylated liposome encapsulated with methotrexate showed improved methotrexate uptake in rats [163]. Au-NPs are often used as they have proven to help transport drugs to induce apoptosis [164].

NPs being carriers also increase the drug stability by preventing the degradation of the encapsulated cargo. Additionally, a large volume of drugs can be encapsulated without any chemical reaction. Dry solid dosage forms are more stable than nanoliquid products [165]. Stabilizers can be used to enhance stability. Yet another way to increase stability is to use porous NPs.

Tumor has unique pathophysiology features such as extensive angiogenesis, flawed vascular architecture and defective lymphatic drainage. The NPs use these features to target tumor tissue. Due to reduced venous return in tumor tissue and meager lymphatic clearance, NPs are effectively retained. This phenomenon is known as EPR. Similarly, by targeting the adjacent tissues, tumor-targeting can be accomplished [166].

NPs can be administered through several routes like oral, nasal, parenteral, intra-ocular etc. NPs have a high surface-to-volume ratio and intracellular uptake. Studies have reported that NPs are more effective than microparticles as drug carriers [167].

### Nanoparticles in Immunotherapy

The immune system sets an important part in the establishment and development of cancer cells. The advancement of immunotherapy has revolutionized cancer therapy. It is found that NPs not only help in target delivery of chemotherapy but can also be used in combination with immunotherapy. There are several approaches in immunotherapy aimed at activating the immune system against cancer cells [168] by “immune checkpoint blockade therapy,” “cancer vaccine therapy,” “chimeric antigen receptor (CAR)-T cell therapy,” and “immune system modulator therapy” [169–171]. NP-based immunotherapy includes “nanovaccines,” “aAPCs (artificial antigen-presenting cells),” and “immunosuppressed TME targeting.”

Nanovaccines specialize in delivering “tumor-associated antigens” and “adjuvants” to antigen-presenting cells, such as dendritic cells (DCs) [172]. Moreover, these can also be employed as adjuvants to enhance “APC antigen presentation” and promote DC maturation that leads to the stimulation of cytotoxic T cells that have anti-tumor function [173, 174]. Liposomes, PLGA NPs, gold NPs are found to have the ability to deliver TAAs into DCs in the cytoplasm [175]. Mesoporous silica, the most used inorganic NP, has exhibited an adjuvant role, leading to immune response stimulation [176]. Artificial APCs interact with MHC-antigen complexes directly which binds to T cells. They also bind to co-stimulatory molecules that bind to co-stimulatory receptors leading to T cell activation [177]. Targeting the immunosuppressed TME is yet another method of using NPs in immunotherapies. This is done by targeting essential cell types in TME such as “tumor-associated macrophages (TAMs),” regulatory T cells, and “myeloid-derived suppressor cells (MDSCs).”

Besides, the combination of chemoimmunotherapy has been demonstrated to be a capable approach in cancer therapy. For instance, a study has shown that co-loading Nutlin-3a, which is a chemotherapeutic agent and cytokine GM-CSF, in “spermine-modified acetylated dextran (AcDEX) NPs” improved cytotoxic CD8( +) T cells proliferation and activated an immune response [178].

“Programmed cell death protein 1 (PD-1)” and “programmed cell death ligand 1 (PD-L1)” are some of the essential immune checkpoints [179]. Hence immune checkpoint inhibitors are used to target these using NPs. According to a study, conventional immune checkpoint inhibitors of PD-L1/PD-1 displayed inconsistent responses. To enhance the chances and bonding of immune checkpoint inhibitors and immune checkpoints, multivalent poly (amidoamine) dendrimers were used. Usage of these dendrimers not only showed enhanced PD-L1 blockade but also showed improved drug accumulation at the tumor site [180].

### Nanoparticles in Cryosurgery

Cryosurgery is an advanced practice of freeze-destroying cancer tissue. Although this is less invasive and causes intraoperative bleeding and postoperative complications, certain drawbacks like inadequate freezing capacity and damage to adjacent cells need to be addressed [181]. The rise of nanotechnology has enabled the use of NPs in cryosurgery.

**Fig. 6:**
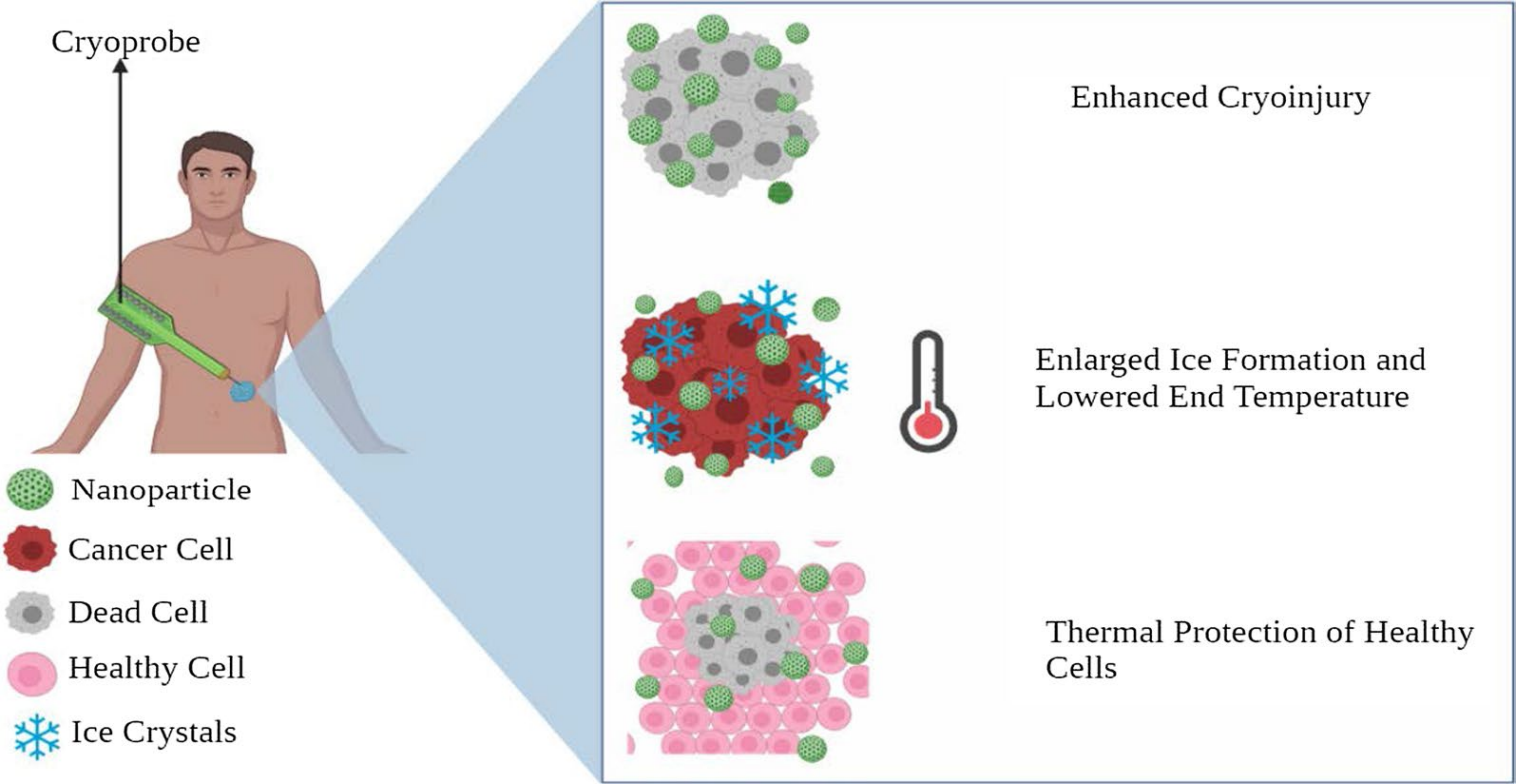
Diagrammatic representation of NPs in cryosurgery

The primary working of nanocryosurgery is introducing NPs with particular properties into the cancer cells and causing freezing [182]. During this process, ice is formed within the cells, which causes damage to it. This is an important process and can be carried out effectively using NPs. The thermal conductivity property of NPs can be exploited, which significantly freeze the tumor tissue and cause tumor damage [183]. Besides, they cool down rapidly, and it is feasible to regulate the “growth direction” and “direction of the ice ball” (Fig. 6).

When the location of the tumor makes it not feasible for cryosurgery or if other adjacent organs are at risk, there are high chances that the freezing can damage healthy tissue. Recently, phase change materials (PMs) made up of NPs are used to protect the adjacent normal healthy tissue during cryosurgery [184]. For instance, liposome-based microencapsulated phase change NPs have shown incredible results in protecting surrounding healthy tissue [185]. These NPs are deemed to possess large latent heat and low thermal conductivity, making them perfect for cryosurgery.

## Significant Challenges in the Clinical Application of Nanoparticles

At present, as nanotechnology has bloomed, the amount of knowledge and research put into nanoparticles has steeply raised. But only a few of them actually make it up to clinical trials. Most of them only halt at in vivo and in vitro stages. Each individual nanoformulation has particular challenges in their clinical translation, but most NPs face similar challenges that can be divided into biological, technological, and study-design related.

Biological challenges include lack of routes of administration, tempering biodistribution, the channel of NPs across the biological barriers, their degradation, and toxicity [186]. NPs are usually injected via intravenous injections directly into the blood, which takes away NPs, making it challenging to stay and interact with the target site. As a result, a high concentration drug is used, which might not provide desired therapeutic effects [187]. However, magnetic NPs can be used to overcome this as many in vivo and in vitro studies have proved the usage of 3D magnetic fields to control the movement of NPs against blood flow. But, the effect of magnetic fields on the human body, crosstalk between magnetic fields, and a large number of NPs has to be researched upon.

Controlling the biological fate of NPs is very hard and needs a lot of focus. Even though NPs are made up of biosafety materials and are modulated accordingly to increase the retention time and half-life, there runs a risk of lung, liver and kidney damage. Some factors that govern toxicity are surface area, particle size and shape, solubility, and agglomeration [188]. NPs have shown greater deposition in the lung with inflammatory, oxidative and cytotoxic effects [189]. Studies reveal that healthy cells often suffer from free radicals generated by NPs [190]. Fabricating NPs with more biocompatible substances like chitosan and materials that disintegrate after near infrared light irradiation may be potential solutions.

Another tricky challenge is avoiding the “mononuclear phagocytic system (MPS).” In biological fluids, NPs adsorb proteins to produce PC, which attacks MPS to uptake NPs. To escape this, NPs have been coated with materials that prevent the formation of the protein corona. However, they have not shown any significant results. Designing NPs that target “macrophages” and using those as new drug vehicles can be pitched to overcome this problem. Currently, preventing macrophage recruitment, depleting and reprograming TAMs, and obstructing “CD47-SIRPα pathways” are commonly used strategies [191].

Technological challenges of NPs include scale-up synthesis, equal optimization, and performance predictions. These are very crucial in safeguarding the clinical success of NPs. Most of the NPs that are used in vivo and in vitro studies are usually produced in minor batches, and scale-up for huge quantities is not constantly feasible given instrumentation and other reasons. The lead clinical candidates that prove to be the best in animal models are not systematically designed optimized. To overcome this, we can use certain methods that can test numerous nanoformulation and by selective iterations selecting a single optimized formulation [192–194]. However, such hits shouldn’t be introduced directly in human testing. Predicting nanoparticle efficacy and performance is hard and replicating the in vivo results in human trials is a herculean task. Computational or theoretical modeling along with experimental results can be designed to imitate physiological tissue and surrounding. For instance, organs-on-chips are being actively studied and can improve NP predictions of efficacy and performance.

Study-design challenges like study size, intent, and timing of NP therapies during the therapy impact significantly during clinical studies. Most of the studies revolve around “cell and animal models” that may not provide comprehensible results in human trials. Therefore, the usage of a single model is tough to imitate natural reactions in the human body. In addition, “models of cancer metastasis” should be actively researched as metastasis is one of the significant properties of cancer. Moreover, *N* = 1 clinical studies will be required if we focus on personalized medicine. This needs to count in many factors such as genetic, environmental, and past medical history. [195, 196]. Another major challenge is that NPs are never used as first-line therapies. Although we have effectively approved nanoformulations, they are usually saved for further treatment if disease progression is found in the clinical trial scenario. Most of the patients have either had progressed on multiple lines of therapies or have gained drug resistance. These situations often skew the clinical trial results and lessen the chance of NP treatment to benefit those who are likely still treatable.

## Conclusion and Future Perspective

Nanotechnology has shown a promising new era of cancer treatment by delivering small molecules for cancer detection, diagnosis, and therapy. Cancer therapies based on the exceptional features of NPs are being vastly used in the clinical setting of several cancer types. NP-based DDS is linked with enhanced pharmacokinetics, biocompatibility, tumor targeting, and stability compared to conventional drugs. Moreover, NPs provide an excellent platform for combination therapy which helps in overcoming MDR. With increasing research, several types of NPs, such as polymeric NPs, metallic NPs, and hybrid NPs, have shown improved efficacy of drug delivery. Researchers must be well attentive to the features of the nominated nanoplatforms and the properties of therapeutic agents. However, there are certain limitations like deficiency of in vitro models that precisely replicate in vivo stage, immunotoxicity, the long-term toxicity, and neurotoxicity. Although “nanovaccines” and “artificial APCs” have proved improved efficacy compared to conventional immunotherapy, the clinical efficacy is substandard. The safety and tolerance of these new modalities should to be inspected. Additionally, developing “immunomodulatory factor-loaded NPs” may advance the efficiency of vaccines for immunotherapy.

This is an emerging area, and it is anticipated that with growth in proteomics research on the “mechanism of cancer origin, MDR, occurrence,” more NP-based drugs can be exploited. Compared to the mammoth amount of investigations, only a few NP-based drugs are actually in use, a few others in clinical trials, and most in the exploratory stage. For rational nanotechnology design, more efforts must be reserved in “understanding toxicity, cellular and physiological factors that regulate NP-based drug delivery, EPR, and PC mechanism” in the human body. Based on the evidence cited above, we presuppose that the revolution in clinical translation for NP-based cancer therapy will be attained with nanotechnology and cancer therapy development.

## Data Availability

Not applicable.
